# Editorial: Community series in recent advances in *Drosophila* cellular and humoral innate immunity: volume II

**DOI:** 10.3389/fimmu.2024.1416296

**Published:** 2024-06-18

**Authors:** Laura Vesala, Dan Hultmark, Susanna Valanne

**Affiliations:** ^1^ Faculty of Medicine and Health Technology, Tampere University, Tampere, Finland; ^2^ Department of Molecular Biology, Umeå University, Umeå, Sweden

**Keywords:** *Drosophila melanogaster*, innate immunity, cellular immunity, humoral immunity, model for human diseases, metabolism, parasitoid wasp, host-pathogen interaction

We are happy to present the Community series in Recent advances in *Drosophila* cellular and humoral innate immunity: Volume II, exploring the advances made in the field since our previous Research Topic in 2019–2020. This Research Topic is comprised of original research and review articles by experts in the field.

Intricacies of especially the cell-mediated innate immune responses are currently under active research in *Drosophila melanogaster* and recent studies have increased our understanding of the functions of *Drosophila* blood cells, the hemocytes, in immune responses and beyond (1–3). Particularly the phagocytic plasmatocytes have proven to be much more diverse than previously acknowledged [reviewed in (4)] and new parallels between plasmatocytes and mammalian monocyte/macrophage lineage have been drawn (5).

Here, the work of several contributors sheds light on immune cell diversity and the regulatory mechanisms that govern their function in developmental and immune contexts. Bazzi et al. discovered a role for the zinc finger transcription factor glial cells missing (gcm) in hemocytes as an anti-inflammatory factor. Loss of *gcm* exacerbates Toll-induced hemocyte activation and differentiation of an immune-activated hemocyte, the lamellocyte, possibly via increase in reactive oxygen species (ROS). Furthermore, their data indicate that hemocyte proliferation can be regulated via the activation of nicotinic acetylcholine receptors expressed in hemocytes, rising interesting questions on the role of neurotransmitter signaling in the control of hemocyte homeostasis. Brooks et al. examined the heterogeneity of plasmatocytes in their response to developmental transitions and apoptotic cells. They show that the phosphatidylserine receptor Simu and Ecdysone receptor signaling influence the identity and function of plasmatocyte subpopulations. Their findings provide insights into hemocyte plasticity and the potential for hemocyte reprogramming in response to environmental challenges. Khalili et al. utilized single cell RNA sequencing to study tumor-associated hemocytes (TAHs). Their work nicely illustrates how TAHs have different characteristics depending on the tumor model and finds similarities to mammalian tumor-associated macrophages. Hemocyte repertoire and functions get even more diverse when other drosophilid species are considered. Cinege et al. studied the multinucleated giant hemocytes in *Drosophila ananassae* and *Zaprionus indianus*. Akin to lamellocytes, these hemocytes encapsulate parasitoid eggs and larvae but resemble human megakaryocytes. Their work gives intriguing insights into the various ways species have adapted to combat pathogenic parasitoids.

Metabolic changes shaping, or dictating, immune cell functions and lineage specification is an active research area especially in vertebrate model systems. Immunometabolism is an increasingly intense field of study also in *Drosophila* (6), see e.g. the recent research by McMullen and coworkers (7). Following on the metabolic processes shaping immune reactions, Darby & Lazzaro review the interactions between innate immunity and insulin-like signaling (IlS) in insects, namely *D. melanogaster* but also *Bombyx mori* and *Anopheles* mosquitos. Insulin signaling and innate immune mechanisms are well conserved from insects to men, and research on the interplay of these processes in insects produces information also applicable to human health. The review concludes that innate immunity and IIS are interconnected, highlighting the energetic demands of an immune response and the role of IlS as a metabolic regulator. Dolezal sums up what is known about metabolic changes occurring in hemocytes during the immune response against parasitoid wasps. A central conundrum is how immune cells balance between generating toxic molecules such as ROS to the extent that kills the parasitoid and, at the same time, protecting the host larva from detrimental effects of ROS. As both generating and eliminating ROS require nicotinamide adenine dinucleotide phosphate (NADP^+^/NADPH), the cyclic pentose phosphate pathway reducing NADP^+^ to NADPH has emerged as a central metabolic pathway in immune-activated hemocytes (lamellocytes). Dolezal further discusses the metabolic intricacies of hemocytes responsible for the encapsulation and poses several questions remaining to be answered. Leitão et al. studied the cost of the immune response against the parasitoids and concluded that the cost comes rather from the direct harm caused by the parasitoid than from mounting the immune response itself, including differentiation and proliferation of hemocytes and the melanization response.

The review by Mpamhanga and Kounatidis discusses the rising global impact of fungal infections, particularly in individuals with chronic and immunosuppressive conditions. It emphasizes the need for advanced research and public health interventions considering the WHO’s fungal priority list. The authors highlight the utility of *D. melanogaster* as a great model organism in the context of studying host-pathogen interactions and immunopathogenesis of fungal diseases, including studying human fungal pathogens and testing antifungal compounds.

Finally, Aalto et al. took advantage of the conservation and tractability of the immune system of *D. melanogaster* and studied the putative anti-inflammatory function of stilbenoids, antioxidant compounds found in plants (8). They discovered that stilbenoids suppress the inflammatory response via dampening the NF-κB-mediated gene expression and that this effect was dependent on the transient receptor potential ankyrin 1 (TrpA1), similarly to mammals. This study demonstrates the potential of *Drosophila* for detailed analysis of the molecular mode of action of putative therapeutic compounds *in vivo*. Recent research shows the power of *Drosophila* in revealing therapeutic targets for cancer (9, 10), rare genetic disorders (11) and neurodegenerative diseases (12), to name a few. It will be exciting to see the innovative approaches taken in the future on various frontiers in drug discovery.

We would like to thank the authors for their contribution to the Research Topic as well as the reviewers for their insightful comments and suggestions. We have no doubt that there are more discoveries to be made regarding the pathways and patterns of immunity in *Drosophila* and are looking forward to the future work done on the findings published in our topic.

## Author contributions

LV: Writing – original draft, Writing – review & editing. DH: Writing – original draft, Writing – review & editing. SV: Writing – original draft, Writing – review & editing.
